# Pennsylvania Hospital Report for the Year 1850

**Published:** 1851-04

**Authors:** 


*Pennsylvania Hospital Report for the Year* 1850.—This well known Insane Asylum, under the management of Dr. Kirkbride, maintains its high reputation. It needs no commendation from us.

